# Functional Analysis Reveals the Regulatory Role of *PpTST1* Encoding Tonoplast Sugar Transporter in Sugar Accumulation of Peach Fruit

**DOI:** 10.3390/ijms21031112

**Published:** 2020-02-07

**Authors:** Qian Peng, Lu Wang, Collins Ogutu, Jingjing Liu, Li Liu, Md. Dulal Ali Mollah, Yuepeng Han

**Affiliations:** 1 CAS Key Laboratory of Plant Germplasm Enhancement and Specialty Agriculture, Wuhan Botanical Garden, The Innovative Academy of Seed Design, Chinese Academy of Sciences, Wuhan 430074, China; 2 University of Chinese Academy of Sciences, 19A Yuquanlu, Beijing 100049, China; 3 Center of Economic Botany, Core Botanical Gardens, Chinese Academy of Sciences, Wuhan 430074, China; 4 Sino-African Joint Research Center, Chinese Academy of Sciences, Wuhan 430074, China

**Keywords:** *Prunus persica*, tonoplast sugar transporter, dCAPS, QTL, fruit sweetness

## Abstract

Sugar content is related to fruit sweetness, and the complex mechanisms underlying fruit sugar accumulation still remain elusive. Here, we report a peach *PpTST1* gene encoding tonoplast sugar transporter that is located in the quantitative trait loci (QTL) interval on Chr5 controlling fruit sucrose content. One derived Cleaved Amplified Polymorphic Sequence (dCAPS) marker was developed based on a nonsynonymous G/T variant in the third exon of *PpTST1*. Genotyping of peach cultivars with the dCAPS marker revealed a significant difference in fruit sugar content among genotypes. *PpTST1* is located in the tonoplast, and substitution of glutamine by histidine caused by the G/T variation has no impact on subcellular location. The expression profile of *PpTST1* exhibited a consistency with the sugar accumulation pattern, and its transient silencing significantly inhibited sugar accumulation in peach fruits. All of these results demonstrated the role of *PpTST1* in regulating sugar accumulation in peach fruit. In addition, cis-elements for binding of MYB and WRKY transcript factors were found in the promoter sequence of *PpTST1*, suggesting a gene regulatory network of fruit sugar accumulation. Our results are not only helpful for understanding the mechanisms underlying fruit sugar accumulation, but will also be useful for the genetic improvement of fruit sweetness in peach breeding programs.

## 1. Introduction

The peach (*Prunus persica* (L.) Batsch) is considered an important economical fruit crop worldwide. Flavor is one of the key factors affecting fruit quality, and it largely depends on soluble sugar and organic acid contents [1]. Sweetness, determined by the level of soluble sugars, is one of the main attributes that influence the degree of consumer satisfaction for peaches [2]. In mature peach fruit, sucrose accounts for over 54% of total soluble sugars, which are mainly stored in the vacuole, occupying up to 90% of the total cell volume [3]. However, the mechanisms underlying sugar accumulation in peach fruit remain largely unknown.

Tonoplast sugar transporters (TSTs) are capable of loading soluble sugars into the vacuole. TSTs belong to a major facilitator superfamily (MFS) that is characterized by 12 transmembrane domains (TMs), with a unique, large central loop [4,5,6]. The first TST was identified in *Arabidopsis*; it was designated tonoplast monosaccharide transporter (TMT) as its lethal mutation caused a significant decrease in monosaccharide accumulation [7]. However, an increasing number of studies are showing that TMTs function as proton-coupled antiporters that are able to transport sucrose into the vacuole [8,9]. Thus, TMTs were renamed as TSTs in a recent study [9].

TSTs have played an important role in sugar accumulation in a variety of crop species. For example, two sorghum TST genes, *SbTST1* and *SbTST2*, show higher levels of expression in the leaf and stem of sweet sorghum, which is rich in soluble sugars when compared to grain sorghum (which mainly accumulates starch) [10]. In rice, *OsTMT1* encoding a tonoplast H^+^/glucose antiporter are highly expressed in vascular tissues, and it is responsible for sugar translocation in both source and sink organs [11]. In *Setaria viridis*, *SvTMTs* associated with soluble sugar accumulation are significantly upregulated in the mature zone of the internode [12]. In the sugar beet, a tonoplast sucrose specific transporter, BvTST2.1, is responsible for sucrose accumulation in taproots [9].

Up until now, TSTs have also been identified in many fruit crops, such as the cucumber [13], citrus [14], pear [15,16], apple [17,18], grape [19,20], watermelon [21], and melon [22]. TSTs have proven to be crucial for sugar accumulation in fruit as well. For example, the *ClTST2* gene is located in a major quantitative trait loci (QTL) for fruit sugar content in watermelon, and its expression shows a well correlation with sugar accumulation in fruit [21]. In *Cucumis melo*, the *CmTST2* gene exhibits higher levels of expression in fruits of high-sugar varieties than in fruits of low-sugar varieties, and its ectopic expression in strawberry and cucumber leads to increased sugar content in fruits [22]. In apples, silencing of *MdTMT1* results in a significant decrease in glucose content [17].

During the past three decades, QTLs for fruit sweetness have been identified in all eight chromosomes of the peach [23,24,25,26,27,28,29]. However, no candidate genes for fruit sweetness have been identified in the peach. Given that sucrose is the predominant sugar in peach fruit, and TSTs are able to act as H^+^/sucrose antiporters, we investigated the TST genes in the peach reference genome [30]. Interestingly, a TST gene (Prupe_5G006300), designated *PpTST1*, is located in a QTL region controlling fruit sucrose content, which accounts for 29% of phenotypic variation [24,25], and overlaps with the *D* locus, harboring a major QTL for fruit acidity [25,31]. Here, functional analysis of *PpTST1* was carried out and the results demonstrate that it is responsible for sugar accumulation in the peach fruit. In addition, a gene-tagged marker was also developed for *PpTST1*. Our results are not only helpful for understanding the complex mechanism underlying sugar accumulation in peach fruit, but will also be useful for genetic improvement of fruit sweetness in peach breeding programs.

## 2. Results

### 2.1. The Expression Profile of PpTST1 Showed a Consistency with the Accumulation Pattern of Soluble Sugars in Peach Fruits

**Figure 1:**
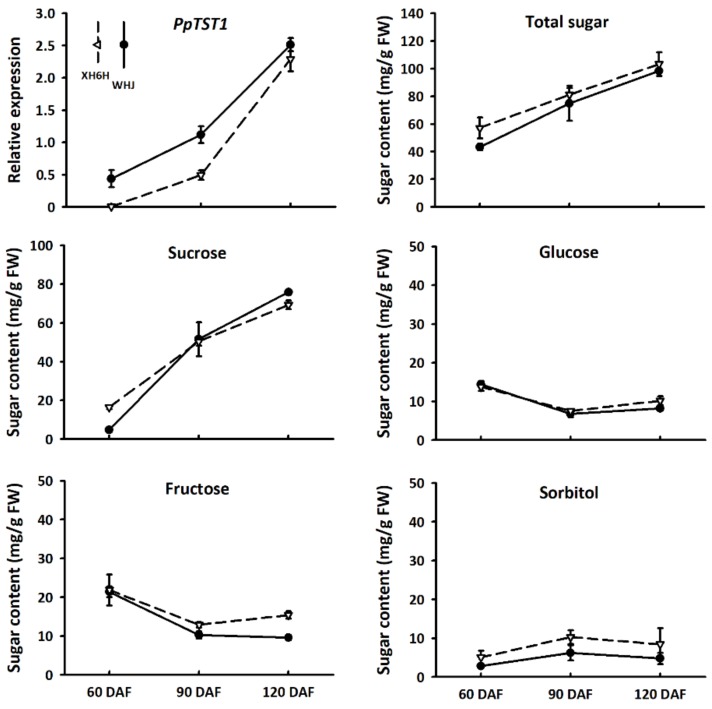
Relative expression of the *PpTST1* gene, and accumulation of sugar components in peach fruits at different developmental stages. DAF, days after full bloom. Error bars represent standard error (SE) of three biological replicates.

To investigate whether *PpTST1* had a potential role in the regulation of sugar accumulation in peach fruit, its expression profile along with sugar accumulation were examined in peach fruit (cvs. Xiahui 6 (XH6H) and Wanhujing (WHJ)) at different stages of development. As a result, *PpTST1* showed an increase in expression levels throughout fruit development, with a peak at the mature stage (120 days after full bloom (DAF)) (Figure 1). Sucrose was the predominant sugar in mature fruits of both XH6H and WHJ, accounting for 67% and 77% of total sugars, respectively. The contents of both sucrose and total sugars showed an increasing trend throughout fruit development. However, the contents of glucose, fructose, and sorbitol showed slight changes throughout fruit development. Overall, the expression profile of *PpTST1* displayed a consistency with the sugar accumulation pattern in peach fruits, suggesting a potential role of *PpTST1* in the regulation of fruit sugar accumulation.

### 2.2. The Impact of PpTST1 on Fruit Sugar Accumulation

**Figure 2:**
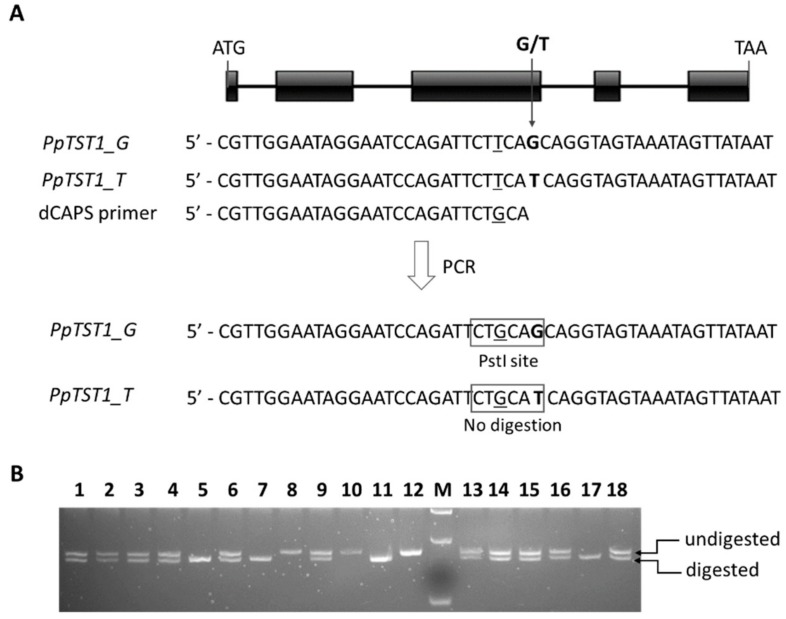
Development and application of gene-tagged molecular marker. (**A**) derived Cleaved Amplified Polymorphic Sequence (dCAPS) marker development for *PpTST1*. The G/T single nucleotide polymorphism (SNP) is highlighted in bold. The underlined character indicates the mismatched base for introducing the dCAPS marker. (**B**) genotyping of peach cultivars with the dCAPS marker. PCR products of each cultivar was digested with PstI and then separated on agarose gel. 1, PANDANA; 2, Diwang 1; 3, Gubatiantao; 4, Hongburuan; 5, 97-1-1; 6, Jinxiayoupantao; 7, Frederica; 8, Hanyin 22; 9, GHETA; 10, Asama Hakuto; 11, Lianhuang; 12, Zheng 97-4-51; 13, 4-3-3; 14, 99-13-9; 15, Xiahui 6; 16, Zhongyoupantao 2; 17, Meiguowanyou; and 18, Ruiguang 19. M, DNA ladder.

Comparison of our previously reported RNA-Sequencing (Seq) data [32] with the peach reference genome revealed a nonsynonymous G/T single nucleotide polymorphism (SNP) in the third exon of the *PpTST1* gene (Figure 2A). To facilitate description, the G and T alleles of *PpTST1* were designated as *PpTST1_G* and *PpTST1_T*, respectively. The G/T SNP was converted into a derived Cleaved Amplified Polymorphic Sequence (dCAPS) marker based on the restriction enzyme PstI. PCR products of the *PpTST1_G* allele contained PstI recognition sequence CTGCAG, while a corresponding non-symmetrical recognition sequence CTGCAT was generated for PCR products of the *PpTST1_T* allele. Thus, the *PpTST1_G* and *PpTST1_T* alleles could be distinguished by two different PCR fragments, with 184 and 211 bp in size, respectively. This dCAPS marker was subject to screen the G/T polymorphism in a collection of 61 peach cultivars. Accessions with homozygous genotypes, G/G or T/T, had single DNA fragments with 184 or 210 bp in size, respectively, whereas, accessions with the heterozygous genotype G/T generated both the 184-bp and 210-bp DNA fragments (Figure 2B). The majority of tested cultivars (82%) belonged to the heterozygous GT genotype, while cultivars with the homozygous G/G or T/T genotypes accounted for 11% and 7% of all tested cultivars, respectively.

**Figure 3:**
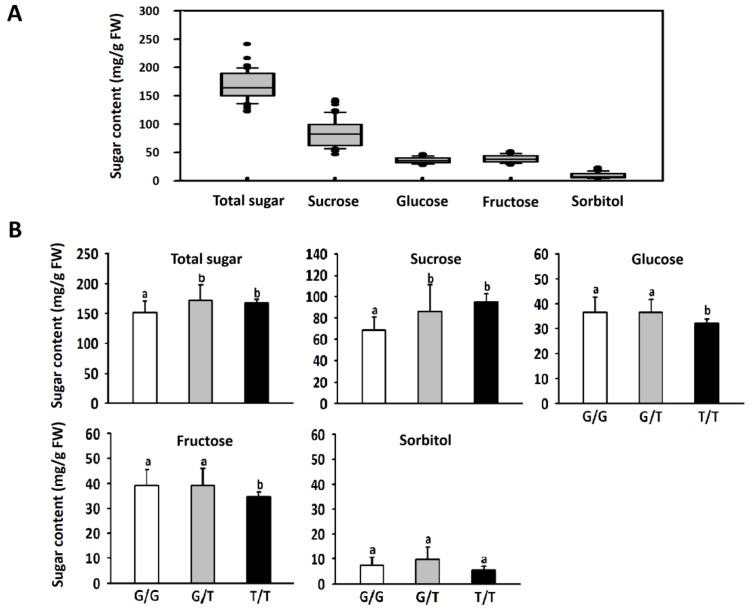
Distribution of soluble sugar content in mature fruits of 61 peach cultivars (**A**), and mean values of sugar contents in mature peach fruits of different genotypes at the G/T locus (**B**). Different lowercase letters indicate significant differences between genotypes (Student’s *t* test at *p* < 0.05).

The sugar content in mature fruits of peach cultivars was measured (Figure 3A). Sucrose was found to be the predominant sugar, and its content ranged from 46.9 to 143.0 mg/g fresh weight (FW), with an average of 84.6 mg/g FW. Total sugar content ranged from 122.4 to 241.0 mg/g FW, with an average of 168.8 mg/g FW. These results indicated that the contents of sucrose and total sugars showed wide variation among cultivars. By contrast, the content of glucose, fructose, and sorbitol exhibited a small degree of variation. As mentioned above, all of the tested cultivars were divided into three genotypes based on the G/T SNP in the third exon of *PpTST1*. Comparison of the sugar content between different genotypes revealed that cultivars with either G/T or T/T genotypes had significantly higher sucrose and total sugar content than cultivars with the G/G genotype (Figure 3B). In contrast, cultivars with either G/G or G/T genotypes showed significantly higher fructose and glucose contents than cultivars with the T/T genotype. However, no significant difference in sorbitol content was observed between different genotypes. Taken together, these results suggest that *PpTST1* has an impact on fruit sugar accumulation in the peach.

### 2.3. Assay for Subcelluar Localization of PpTST1 in Tobacco Leaves

**Figure 4:**
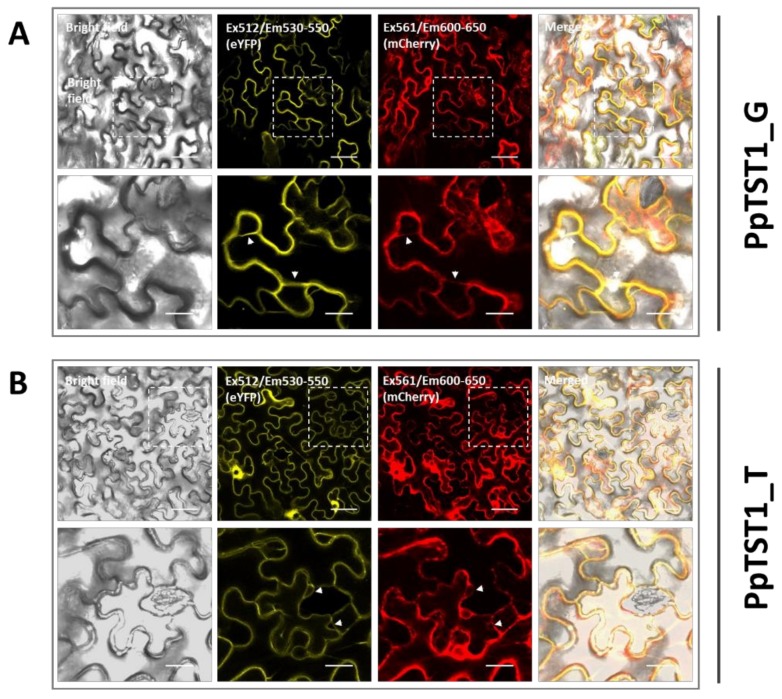
The subcellular localization of PpTST1 in tobacco leaves. Co-expression of pMDC83-eYFP-PpTST1_G (**A**) and pMDC83-eYFP-PpTST1_T (**B**) with tonoplast marker vac-rk CD3-975 in bright field, YFP channel, mCherry channel, and merged channel, respectively. Bottom lane are the enlarged images of the upper lane. The white arrow indicates vacuole membrane. Upper and bottom lane scale bars are 50 and 20 µm, respectively.

Interestingly, the G/T variant in the third exon of *PpTST1* determines a histidine (His) to glutamine (Gln) substitution in the seventh transmembrane domain that was predicted using the Protein Homology/analogY Recognition Engine V 2.0 [33] (Figure S1A,B). To determine whether the His → Gln substitution had an effect on subcellular localization, full-length coding sequences of *PpTST1_G* and *PpTST1_T* were individually fused with yellow fluorescent protein (YFP) to generate *PpTST1_G-YFP* and *PpTST1_T-YFP* constructs under the control driven by the CaMV 35S promoter, respectively. These two constructs were transiently expressed in leaves of *Nicotiana benthamiana*. Both PpTST1_G-YFP and PpTST1_T-YFP fusion proteins were found to reside in the tonoplast (Figure 4). To clarify the tonoplast location of the proteins from two allele gene, co-localization was conducted using the standard vacuolar membrane marker Vac-rk CD3-975 [34]. The YFP fluorescence of PpTST1-YFP was completely merged with mCherry fluorescence of the tonoplast marker Vac-rk CD3-975 (Figure 4A,B). Altogether, these results showed that two alleles of *PpTST1* are both located in the tonoplast, and the G/T SNP in third exon had no effect on subcellular localization.

### 2.4. Transient Silencing of PpTST1 Inhibits Sugar Accumulation in Peach Fruit

**Figure 5:**
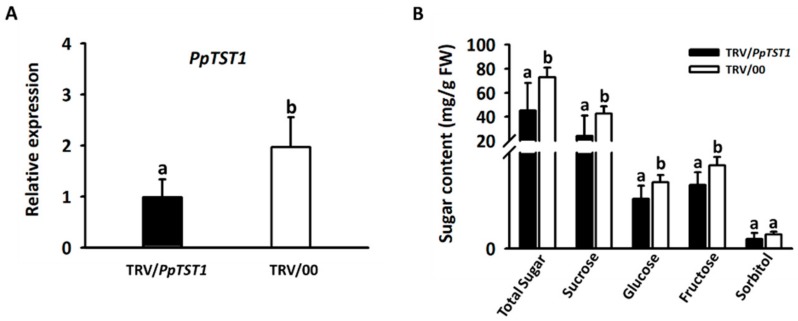
Functional analysis of the *PpTST1* gene using the virus-induced gene silencing (VIGS) system in peach fruits. (**A**) expression of *PpTST1* in peach fruit injected with pTRV2-PpTST1/pTRV1 and pTRV2/pTRV1 (control), respectively. (**B**) sugar accumulation in peach fruit injected with pTRV2-PpTST1/pTRV1 and pTRV2/pTRV1, respectively. Different lowercase letters indicate significant differences between fruits injected with pTRV2-PpTST1/pTRV1 and pTRV2/pTRV1 (Student’s *t* test at *p* < 0.05). pTRV2-PpTST1/pTRV1 and pTRV2/pTRV1 are indicated with TRV/*PpTST1* and TRV/00, respectively. Error bars represent standard error (SE) of three biological replicates.

The role of *PpTST1* in regulating fruit sugar accumulation was also validated using the virus-induced gene silencing (VIGS) system. Specific cDNA sequences covering the end of exon 2 and beginning of exon 3 were amplified and inserted into the pTRV2 vector to generate a pTRV2-PpTST1 construct. *Agrobacterium tumefaciens* harboring pTRV1 and pTRV2-PpTST1 was injected into fruit at the ripening stage. Real time quantitative PCR (RT-qPCR) analysis indicated that the expression level of *PpTST1* was significantly decreased by 50% in infiltration areas 7 days after transformation with pTRV1 and pTRV2-PpTST1, compared with infiltration areas transformed with pTRV1 and pTRV2 (Figure 5A). High performance liquid chromatography (HPLC) analysis further revealed that silencing of *PpTST1* reduced the contents of sucrose, glucose, fructose and total sugars by 43.2%, 25.0%, 23.6%, and 38.1%, respectively. However, no significant decrease was observed for sorbitol content (Figure 5B). Taken together, these results demonstrated that the *PpTST1* gene is involved in the regulation of sugar accumulation in peach fruit.

### 2.5. Multiple Cis-Elements Are Present in the Promoter Region of PpTST1

The 2-kb upstream of the start codon of *PpTST1* in XH6H was isolated and annotated using the program PlantCARE [35]. The promoter sequence contained various putative cis-elements involved in response to stress, hormone signaling, and light response (Table S2). In addition, six MYB-related DNA binding motifs and a W-box motif, the highly conserved binding site for WRKY TFs, were also identified. These results indicated that the expression of *PpTST1* might be regulated by TFs, such as WRKYs and MYBs, and in response to various environmental stimuli, which is consistent with the finding that fruit sugar accumulation is controlled by environmental and genetic factors [17,21].

## 3. Discussion

Sweetness is one of the most important components of fruit taste quality [1]. Soluble sugars that are primarily stored in the vacuole contribute to the sweetness of fruits [3]. Recent studies show that TSTs are responsible for accumulation of the predominant sugar, sucrose, in fruits or roots of many crops, such as the melon [22], watermelon [21], and sugar beet [9]. Likewise, sucrose also represents the predominant sugar in peach fruit. Up until now, genetic mapping has revealed a total of six QTLs for fruit sucrose content, which are located on chromosomes 1, 3, 5, 6, 7, and 8, respectively [24,25,26,27,36,37]. There are four copies of TST genes in the peach genome [38]. Of these four *PpTSTs*, two, *Prupe_7G185700* and *Prupe_7G186000*, are clustered on Chr7, while the other two, *PpTST1* and *Prupe_8G180600*, are located on Chr5 and Chr8, respectively. The two clustered *PpTSTs* are located in a QTL interval for sucrose content [26,27], but they show extremely low levels of expression in fruits [38]. *PpTST1* is also located within a QTL confidence interval for fruit sucrose content, and its transcript is the most abundant in fruits during the ripening stages compared with other *PpTSTs,* based on our previous RNA-Seq data [32]. Subcellular localization analysis shows that PpTST1 is located in the vacuolar membrane. Transient silencing of *PpTST1* significantly inhibits sucrose accumulation in peach fruit. All of these results suggest that only *PpTST1* out of the TST gene family in the peach is likely responsible for sucrose accumulation in fruit, and it is a strong gene candidate in the QTL interval on Chr5 that controls sugar content of peach fruit.

It is worth noting that silencing of *PpTST1* in peach fruit also inhibited hexose accumulation, suggesting that it may also have influence on hexose accumulation. This finding is consistent with previous reports that modification of expression of *CmTST2* and *ClTST2* results in changes in the accumulation of both sucrose and hexose in the melon and watermelon, respectively [21,22]. TSTs function as antiporters that import sugars in exchange for protons, with a 1:1 stoichiometric ratio [8,39]. Thus, it is worthy of further study to ascertain the potential impact of the *PpTST1* gene on fruit acidity and/or the sugar-acid ratio as it is located in the major *D* locus for fruit acidity [25,31]. In addition, the *PpTST* gene, *Prupe_8G180600*, was found to be highly expressed in peach fruit [32,38]. Given that TSTs are capable of loading sucrose and/or hexose into the vacuole [8,39], it is reasonable to speculate that *Prupe_8G180600* may be involved in hexose accumulation in peach fruit.

It has been reported that expression levels of TSTs are well consistent with sugar accumulation in many crops [9,21,22]. Here, our results show that the expression profile of *PpTST1* is also consistent with the fruit sugar accumulation pattern in the peach. These results suggest that the TST genes appear to be regulated primarily at the level of transcription. A nonsynonymous G/T variant in the third exon of *PpTST1*, which determines a His to Gln substitution, is located at the seventh transmembrane domain (Figure S1A). However, our study shows that this variant has no impact on subcellular location. Thus, the G/T SNP is unlikely a causal mutation, although its genotypes are related to fruit sugar accumulation. However, it cannot be ruled out that the His to Gln substitution might affect the substrate specificity of PpTST1 as transmembrane domains are known to contribute to sugars translocation across vacuolar membrane [40,41].

*Cis*-elements, abscisic acid-responsive element (AREB) [17] and W box [21], in the promoter region of TSTs have been reported to play import roles in regulating fruit sugar accumulation. In this study, a variety of putative cis-elements, such as W box, hormone and light response motifs, and MYB binding sites, are also identified in the promoter sequence of *PpTST1*. Hence, it is worthy of further study to ascertain whether TFs, such as MYBs and WRKYs, are involved in the regulation of the *PpTST1* transcription, which forms a gene regulatory network controlling sugar accumulation in peach fruit.

## 4. Materials and Methods

### 4.1. Plant Materials

Peach (cv. Jinxiu) used for the analysis of tobacco rattle virus (TRV)-mediated virus-induced gene silencing (VIGS) is maintained in the orchard at the Institute of Fruit and Tea, Hubei Academy of Agricultural Sciences, Wuhan, China, while other cultivars are maintained in the peach field at Northwest Agriculture and Forestry University, Yangling, Shaanxi, China. Young leaves were collected in the spring season, and fruit samples were collected at a mature stage. Maturity date was estimated according to previous records, along with fruit that no longer increased in size, softened, easily detached, and showed disappearance of the green skin background color. In addition, two cultivars, Xiahui 6 and Wanhujing, were chosen for gene expression profiling analysis due to their similar duration to maturity. Fruit samples were collected at the following three stages: pit hardening, the second exponential growth, and mature stage, corresponding to 60, 90, and 120 days after full bloom (DAFB), respectively. All fruit samples were peeled and cut into pieces, frozen in liquid nitrogen, and then stored at −80 °C until use.

### 4.2. DNA Extraction and dCAPS Marker Development

Genomic DNA was isolated following a previous report [42]. To identify DNA sequence polymorphism in *PpTST1*, our previously reported RNA-Seq data of peaches [32] were compared with the peach reference genome [30]. A single nucleotide polymorphism (SNP) was detected in the coding region. Subsequently, the web-based free program dCAPS Finder 2.0 [43] was used to convert the SNP into a derived Cleaved Amplified Polymorphic Sequence (dCAPS) marker along with an appropriate restriction enzyme. Primers of the dCAPS marker were designed using online Primer Premier 5 (http://www.premierbiosoft.com/primerdesign/). PCR amplification was conducted in a mixture containing 5 μL 10× PCR buffer, 4 μL 25 mM MgCl_2_, 1 μL 10 mM deoxynucleotide triphosphates (dNTPs), 1 μL 10 μM of each forward and reverse primer, 1U Taq DNA polymerase, and 40 ng DNA template. The PCR program was set as follows: 95 °C for 5 min; 38 cycles of 95 °C for 35 s, 52 °C for 35 s and 72 °C for 2 min 15 s; followed by a final extension at 72 °C for 30 min. Amplified PCR products were digested using a restriction enzyme (Thermo Fisher Scientific, Waltham, MA, USA) and then separated by electrophoresis on 4% agarose gel.

### 4.3. RNA Isolation and RT-qPCR Analysis

Total RNA extraction was performed with the RNAprep Pure Plant kit (Tiangen, Beijing, China) according to the manufacturer’s instructions, and DNase I (Takara, Dalian, China) was used to remove genomic DNA contamination. Preparation of cDNA templates was carried out with TransScript One-Step gDNA Removal and cDNA Synthesis SuperMix (TRANS, Beijing, China) following the manufacturer’s instructions. Real-time quantitative PCR (RT-qPCR) was conducted according to our previously reported protocol [32]. A previously reported actin gene *TEF2* [44] was used as a constitutive control, and a negative control for each sample was also performed. Each sample contained three biological replicates. Primer sequences are listed in Table S1.

### 4.4. Measurement of Sugar Content and Composition in Peach Fruit

Fruit samples were ground into powder in liquid nitrogen using an A11 basic Analytical mill (IKA, Darmstadt, Germany). One gram of powder was dissolved in 6 mL of deionized water. After ultrasonic treatment for 15 min, the mixture was centrifuged at 5000× *g* for 15 min. The supernatants were collected and passed through a filter with a pore size of 0.22 μm. The filtered supernatants were subject to measure sugar content and composition, with high-performance liquid chromatography (HPLC, Agilent 1260 Infinity, Agilent, Waldbronn, Germany), following our previously reported method [45].

### 4.5. Subcellular Localization Analysis of PpTST1 in Tobacco Leaves

The pMDC83 plasmid used in this study was modified. Briefly, pMDC83 was digested with two restricted enzymes, PmeI and EcoRI, to remove a fragment containing a CaMV 35S promoter, WG Gateway cassette (attR2, ccdB, cmR, attR1 orientation), and green-fluorescent protein (GFP) cassette. Meanwhile, the pFGC-eYFP plasmid was also digested with PmeI and EcoRI, and a small fragment of the T-DNA region containing CaMV 35S promoter, eYFP and OCS terminator was collected. This small fragment was then ligated with the digested pMDC83, resulting in a modified vector, designated pMDC83-eYFP. The entire coding DNA sequence (CDS) without the stop codon of the *PpTST1* gene was inserted into pMDC83-eYFP to generate expression construct. The expression vector was transformed into the *A. tumefaciens* strain GV3101 via the heat shock method and then infiltrated into tobacco leaves. Fluorescence was detected 3 days after infiltration using the confocal microscope (TCS SP8, Leica, Microsystems, Wetzlar, Germany). The sequences of primers used for expression vector construction are listed in Table S1.

### 4.6. Functional Analysis of PpTST1 Using the VIGS System in Peach Fruit

A 350-bp partial cDNA fragment of *PpTST1* was amplified and inserted into the *Eco*RI and *Bam*HI site of the pTRV2 vector with an In-Fusion^®^ HD Cloning Kit (Takara) according to the manufacturer’s instructions, resulting in a recombinant construct pTRV2-PpTST1. The pTRV2-PpTST1 vector and two entry vectors, pTRV1 and pTRV2, were individually transferred into the *A. tumefaciens* strain GV3101 using the heat shock method. Transformants were grown on LB agar plates containing 50 mg/L rifampicin, 50 mg/L gentamycin, and 50 mg/L Kanamycin at 28 °C for 3 days. Positive *Agrobacterium* cells were inoculated into LB liquid medium, incubated overnight at 28 °C, and then collected by centrifuging at 4000× *g* for 15 min. The pellet was resuspended in infiltration buffer (50 mM MES, 5 mg/mL d-glucose, 2 mM Na_3_PO_4_, 100 μM acetosyringone), and then diluted to an optical density at 600 nm (OD) _600 nm_ of 0.8. *Agrobacterium* cultures containing pTRV1 were mixed with *Agrobacterium* cultures containing either pTRV2-PpTST1 or pTRV2 in a ratio of 1:1. The infiltration buffer was injected into detached peach fruits at the ripening stage using 1 mL syringes without the needle. The injected fruits were placed in a growth chamber at 25 °C under a 16-h light/8-h dark photoperiod. Peach fruits were subject to RT-qPCR analysis and sugar content measurement 9 days after infiltration. The sequences of primers used for expression vector construction and RT-qPCR are listed in Table S1.

## 5. Conclusions

The *PpTST1* gene in the *D* locus of the peach that encodes a tonoplast sugar transporter, and is a strong candidate that is responsible for fruit sugar accumulation. Further studies are needed to address whether *PpTST1* has a potential effect on fruit acidity and/or the sugar-acid ratio, and whether its expression is regulated by TFs, such as MYBs and WRKYs. Our results are helpful for both understanding the mechanisms underlying fruit sugar accumulation and genetic improvement of fruit sweetness in peach breeding programs.

## Supplementary Materials

Supplementary Materials can be found at https://www.mdpi.com/1422-0067/21/3/1112/s1.
