# Supplementary material for: Functional Analysis Reveals the Regulatory Role of PpTST1 Encoding Tonoplast Sugar Transporter in Sugar Accumulation of Peach Fruit
Source: Int J Mol Sci. 2020 Feb 7;21(3):1112. doi: 10.3390/ijms21031112 (PMC7038102; doi:10.3390/ijms21031112)
Supplement: Supplementary file 1 [file ijms-21-01112-s001.pdf]

Table S1. DNA sequences of primers used in this study.

Gene	Primer (5'→ 3')		Note
	Forward	Reverse	
PpTST1	TTTACATCTCTAGGACTGGCAGGA	CGTTGGAATAGGAATCCAGATTCTGCA	dCAPS
TEF2	GGTGTGACGATGAAGAGTGATG	TGAAGGAGAGGGAAGGTGAAAG	RT-qPCR
PpTST1	CATGGATATCTCTGGTAGAAGGACG	CAGAGGCCACGTACCCTTGTC	RT-qPCR
PpTST1	AATTACATTTACAATTACGATGAGGGGAGCTGT GATGGTGGCTATTG	CTCCTCGCCCTTGCCCATCTCACTTTTGGCTGC GGCAAC	Subcellularl ocation
PpTST1	GTGAGTAAGGTTACCGAATTCCCTGATCGTCAG CAAGTTCA	CGTGAGCTCGGTACCGGATCCATTAAATACTC TTCCACAGT	VIGS

Table S2. Identification of *Cis*-elements in 2kb upstream region of *PpTST1*.

Cis- Element	Motif	Position	Function
AAGAA-motif	GAAAGAA	−1010(+), −1432(−)	Unknown
AP-1	TGAGTTAG	−1883(+)	Stress response element
ARE	AAACCA	−23(+), −90(+), −1054(−), −1243(+)	*Cis*-acting regulatory element essential for the anaerobic induction
as-1	TGACG	−1372(+)	SA-responsive element
Box 4	ATTAAT	−1921(−)	Part of a conserved DNA module involved in light responsiveness
Box III	atCATTTTCACt	−71(−)	Protein binding site
CAAT-box	CCAAT/CAAT/CAAAT	−49(−), −83(+), −125(+), −141(+), −153(+), −176(+), −283(+), −300(−), −312(+), −399(+), −423(+), −506(−), −514(+), −534(+), −543(−), −571(+), −591(+), −600(−), −628(+), −648(+), −723(−), −831(−), −882(+), −897(−), −942(−), −953(+), −998(+), −1029(+), −1048(+), −1280(+), −1462(−), −1497(+), −1513(−), −1534(−), −1798(+), −1968(−)	Common *cis*-acting element in promoter and enhancer regions
CGTCA-motif	CGTCA	−1372(−)	MeJA-responsive element
GARE-motif	TCTGTTG	−1810(+)	Gibberellin-responsive element
HD-Zip 1	CAAT(A/T)ATTG	−953(+)	Element involved in differentiation of the palisade mesophyll cells
MBS	CAACTG	−1208(−), −1325(−), −1368(−), −1719(−)	MYB binding site involved in drought-inducibility
MYB	CAACAG	−1811(−), −1869(−)	MYB binding site
Myc	TCTCTTA	−1443(+)	Unknown
P-box	CCTTTTG	−1852(+)	Gibberellin-responsive element
STRE	AGGGG	−1782(−)	Stress response element
TATA-box	TATAA	−39(−), −166(−), −250(−)	Core promoter element around -30 of transcription start
TCCC-motif	TCTCCCT	−1828(−)	Part of a light responsive element
TCT-motif	TCTTAC	−1445(+)	Part of a light responsive element
W box	TTGACC	−307(+)	WRKY binding site
WUN-motif	AAATTTCTT	−195(+)	Stress response element

A

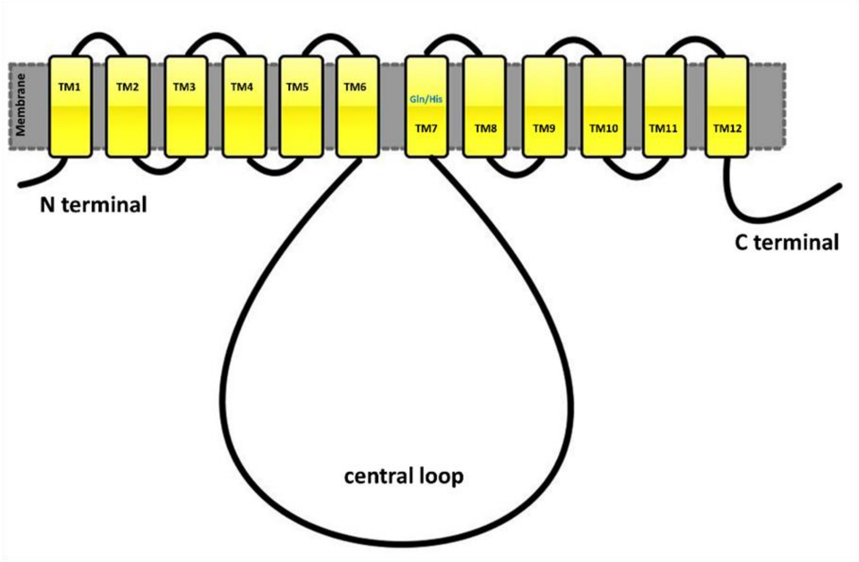

B

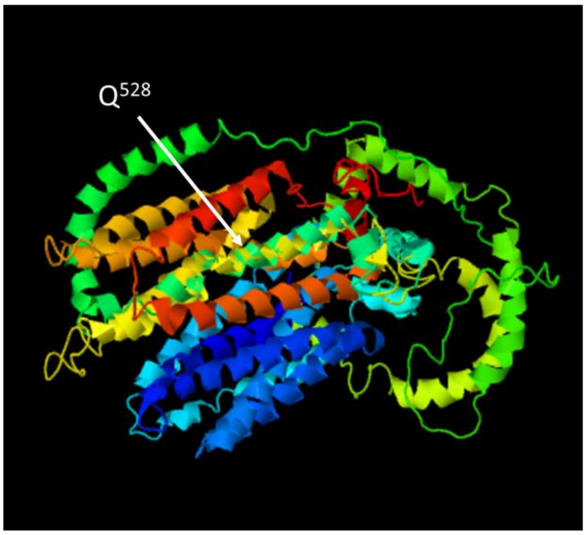

Figure S1. The predicted structure of PpTST1. (**A**), The predicted schematic topology of PpTST1. TM, transmembrane domain. Blue character Gln/His indicates one amino acid substitution resulting from a G/T SNP. (**B**), The tertiary structure of PpTST1 predicted using Protein Homology/analogY Recognition Engine V 2.0 [45]. Q^{528} indicates Gln which could be replaced with His resulting from a G/T SNP.
